# Identification of typical marker proteins of *Treponema pallidum* in compact human bone using morphological and biochemical techniques

**DOI:** 10.1038/s41598-025-12970-z

**Published:** 2025-08-06

**Authors:** Tyede H. Schmidt Schultz, Michael Schultz

**Affiliations:** Department of Anatomy and Cell Biology, University Medical Center Goettingen, Kreuzbergring 36, 37075 Goettingen, Germany

**Keywords:** Bone histology, Microscopy, Paleopathology, Paleoproteomic, *Treponema pallidum*, Biochemistry, Biological techniques, Anatomy, Diseases

## Abstract

In ancient human compact bone tissue, we can present the identified marker proteins of *Treponema pallidum*, the lipoproteins 47 kDa, 17 kDa and 15 kDa in three adult individuals from Austria (thirteenth–seventeenth century CE), and in a 5 to 6-years-old child from Germany (seventeenth–nineteenth century CE). These three identified lipoproteins are also used to diagnose syphilis in current medicine. The individuals selected for this study predominantly exhibit the macroscopic and microscopic features of treponemal disease. However, the result of the proteomic analysis can confirm the diagnosis of treponematosis without any doubt. The extracellular matrix (ECM) proteins in well-preserved ancient human compact bone are still tightly bound to hydroxyapatite and/or collagen. With our solubilization technique, the entrapped ECM proteins are solubilized and identified with special antibodies in Western blot. Our techniques open up more possibilities to diagnose also other infectious diseases and tumors in humans who lived many thousands years ago.

**Supplementary Information:**

The online version contains supplementary material available at 10.1038/s41598-025-12970-z.

## Introduction

Mineralized bone provides an excellent system for the sequestration and subsequent immobilization of extracellular matrix (ECM) proteins, which are normally soluble in physiological fluid. This junkyard-like property of bone can be very beneficial when the bound ECM proteins take advantage of the tissue, either in its normal physiology or under disease-induced stress^1,2^. Many proteins, whether locally or exogenously produced, are bound to bone hydroxyapatite and to collagen during their lifespan^3^. The conditions in the compact bone substance are characterized by the relatively low content of water and degradation enzymes^4^. A chemical advantage is that the majority of non-collagenous proteins (NCPs) contains a high density of aspartic acid and glutamic acid residues. The acid residues have a high affinity for calcium ions due to their charged carboxyl groups^5^. The acidic amino acids also bind to collagen^6^ and this binding to hydroxyapatite and collagen enables NCPs to be more or less at the same place after death as in lifetime. These prerequisites enabled us to identify typical bone proteins and also disease markers in ancient human bone. In addition to the typical bone proteins osteonectin, osteopontin and osteocalcin^7^, growth factors such as morphogenetic protein-2, tumor necrosis factor-a, insulin growth factor- II^8^ immunoglobulin (IgG) are detected^7^ IgG is the main type of antibody and is found in equal amounts in blood and extracellular fluid as ECM of bone^9^. Our oldest paleoproteomically examined bone, which was examined with the technique presented here, comes from a three million-years-old gomphothere proboscidean (*Anancus avernensis*). In this case, we were able to detect osteonectin, osteopontin and bone morphogenetic protein-2 in the Western blot using specific antibodies^10^.

Human diseases are as old as human history. In paleopathology, the science dealing with diseases in ancient cultures, macroscopic, low power and reflected light microscopic and often radiological techniques are routinely used. However, they do not always lead to a convincing diagnosis. For this reason, plain and polarized transmitted light microscopy^11^ and biochemical techniques such as proteomics^12^ were used in combination. In protein biochemistry of human bone, it is possible to differentiate between the various protein disease markers that are built up by the organism of the diseased individual or by typical proteins that are part of the pathogen. An example of the successful detection of a membrane protein from *Mycobacterium tuberculosis* in the ECM of ancient bone are the proteins antigen 85 (Ag85)^12^ and early secreted antigenic target 6 (ESAT-6)^13^.

In tumours, the organism itself mainly forms the so-called disease markers. An example for the detection of a tumour marker is the prostate-specific antigen (PSA), a secretory protein of the human prostate of ~ 30kDa^14^. PSA in complexed form with α1-antichymotrypsin (PSA-ACT) was detected in a bone from a Scythian king (Arzhan in Siberia, Russia), who lived around 700 before Common Era (BCE)^15^. The detection of mainly complexed PSA means a higher malignancy of prostate cancer (CA)^16^.

Syphilis is one of the oldest recognized sexually transmitted infections in humans and the incidence is increasing in many parts of the world. At the beginning of the twentieth-century the bacterium, a spiral shaped spirochete, *Treponema pallidum*, were first described by F. Schaudinn and E. Hoffman^17^. *T. pallidum* is a fastidious, microaerophilic spirochete that has three subspecies: Subspecies *pallidum* (venereal syphilis), subspecies *endemicum* (bejel*/*endemic syphilis) subspecies *pertenue* (yaws)^18^. These three subspecies cause bone changes. Although subspecies differ pathogenically, they are > 95 homologous by DNA-DNA hybridization^19^ and serologically indistinguishable^20^.

After 1970, the application of transmission electron microscopy, biochemical techniques and molecular biology have led to increase the understanding of the morphology, in vivo and in vitro growth characteristics and metabolism of *T. pallidum.* The identification of *T. pallidum* began about 1970 with the application of electrophoretic techniques; based on the sodium dodecylsulphate polyacrylamide gel electrophoresis (SDS-PAGE) results of the *T. pallidum* protein pattern was described^19^. In this publication, we present the identification of typical *T. pallidum* proteins in archaeologically recovered human bone.

## Results

### Morphological features observed

**Fig. 1:**
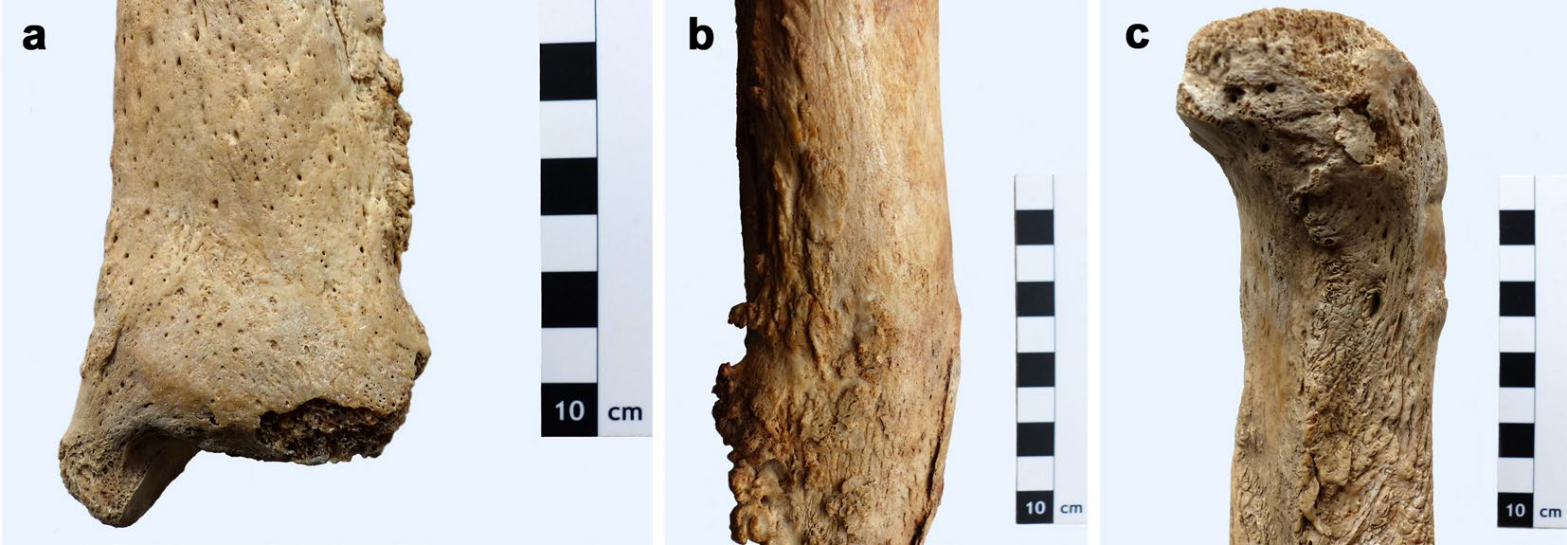
Macroscopic changes of chronic inflammatory bone disease caused by treponematosis. Macroscopic images of the syphilitic tibiae from Klosterneuburg presenting the features of chronic bone inflammation. (**a**) Adult Individual E-4, detailed view on the slightly thickened distal end of the shaft of the left tibia: comparatively smooth and slightly porous, waxy appearance of the external bone surface; (**b**) adult Individual E-5, left tibia: small, irregularly formed, exostotic muscle and tendon attachments in the middle of the shaft; (**c**) adult Individual E-6, right tibia: crest-like new bone formations in the proximal end of the bone shaft.

**Fig. 2:**
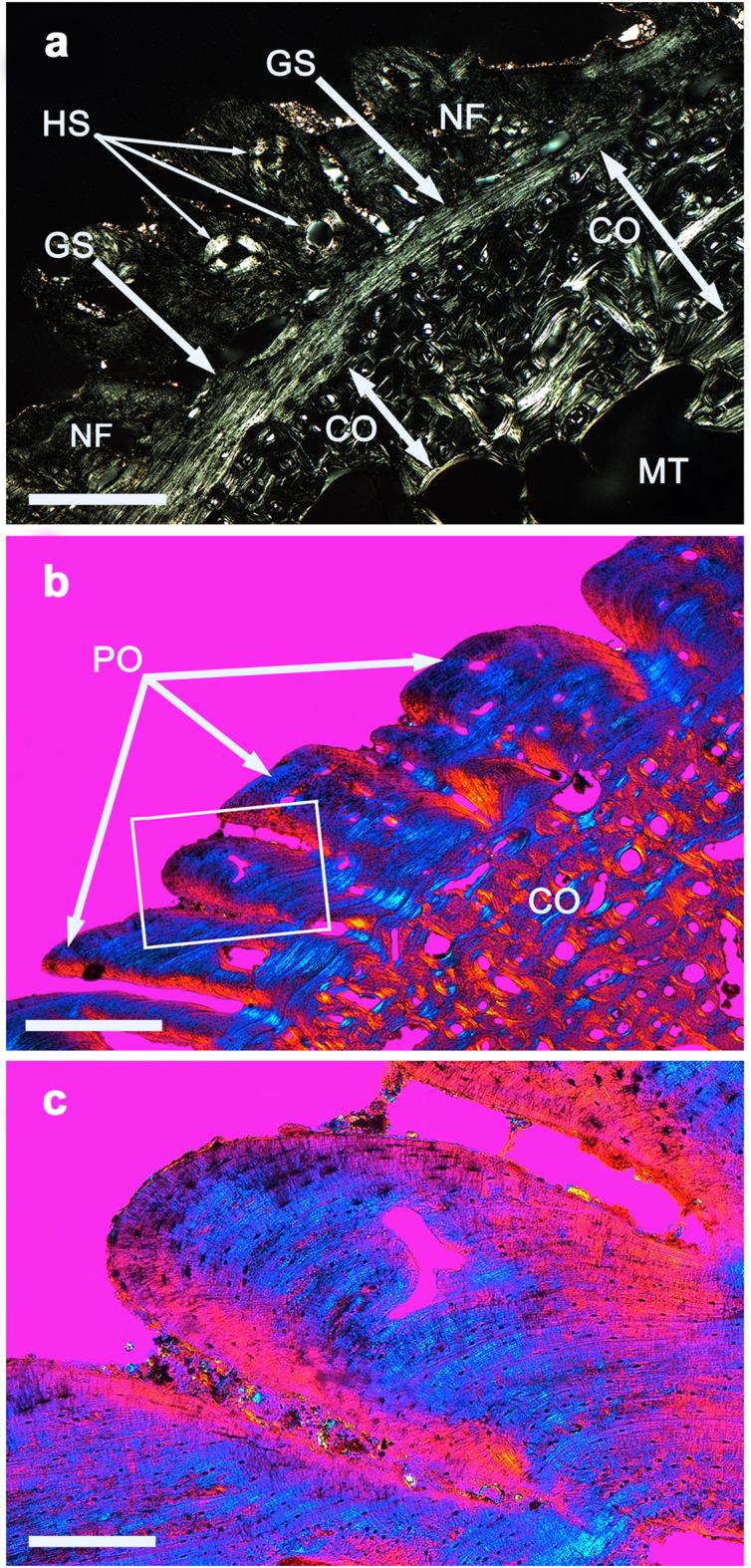
Microscopic changes of chronic inflammatory bone disease caused by treponematosis. Microphotographs of undecalcified thin-ground sections (thickness 50 µm). Cross sections of the right tibia of Individual E-6 viewed. (**a**) Cross section viewed through the microscope in polarized transmitted light. CO = compact, original bone substance that is already beginning to undergo remodeling due to inflammation; HS = Haversian Systems in the external, newly built compact bone; GS = grenzstreifen; MT = medullary tube; NF = newly formed bone. Magnification 25×. Bar 1 mm. (**b**) Cross section viewed through the microscope in polarized transmitted light using a hilfsobject red 1st order (quartz) as compensator. PO = polsters; CO = compact, original bone substance that is already characterized by hypervascularization (blood vessel canals) and some resorption cavities caused by inflammation. Magnification 25x. Bar 1 mm. The rectangular frame outlines the area shown in partial (c). (**c**) Detail from (b). Polster. Magnification 100×. Bar 250 µm.

Macroscopically, the tibiae of the three adult cases from Austria show the characteristic features of chronic inflammatory bone disease (Table 1, 3-5; Fig. 1). The clearly thickened bone shafts have predominantly smooth to slightly porous, partially doughy to waxy surfaces (Fig. 1a). The morphological features indicate a slowly developing process that occurs without dramatic bone dissolution. There are no cloacae, fistula, involucrum and sequester. Muscle and tendon attachments are rough and have small, exostotic (Fig. 1b), sometimes crest-like new bone formations (Fig. 1c) which, however, occur in specific and non-specific osteomyelitis. The light microscopic examination of thin-ground sections showed that the samples selected for proteomic analysis were very well preserved in their internal structure (e.g., collagen) (Fig. 2). The micropathological analysis revealed a grenzstreifen (GS) which represents the remnants of the external circumferential lamellae, and polsters (PO), which in the polarizing microscope consist of parallel arranged collagen fiber bundles, which, in a wavy manner, form the external bone surface as a newly grown product of a slowly progressing chronic inflammation (Fig. 2). Therefore, these three cases most likely represent the presence of non-gummatous treponemal disease. In the 5 to 6-years-old child from Tasdorf (Table 1, 6), there was indication of a very likely congenital syphilitic disease due to mineralization disorders in the sense of a mulberry molar^21^.

**Table 1:** Ancient human bone samples examined for treponematoses.

Geographical location/origin	Individual/burial	Sex	Age	Sampled bone	Dating	Storage conditions
(1) The pathological-anatomical collection of the Natural History Museum Vienna in the Narrenturm (“Fools’ Tower”)	MN 18,560	Unknown	62 years	Right tibia	25.11.1919	Museum specimen
(2) The pathological-anatomical collection of the Natural History Museum Vienna in the Narrenturm (“Fools’ Tower”)	MN 1582	Male	50 years	Left tibia	18.05.1834	Museum specimen
(3) Charnel house of the parish church of St. Martin, Klosterneuburg, Lower Austria (Austria)	Ind. E4	Unknown	Adult	Left tibia	Thirteenth–seventeenth century CE	Charnel house, dry storage condition
(4) Charnel house of the parish church of St. Philipp and St. Jakob, Zellerndorf, Lower Austria (Austria)	Ind. E5	Unknown	Adult	Left tibia	Fourteenth–seventeenth century CE	Charnel house, dry storage condition
(5) Charnel house of the parish church of St. Martin, Klosterneuburg, Lower Austria (Austria)	Ind. E6	Unknown	Adult	Right tibia	Thirteenth–seventeenth century CE	Charnel house, dry storage condition
(6) Cemetery of the parish church, of Tasdorf-Rüdersdorf, near Berlin, Brandenburg (Germany)	Burial 205	Female	Infans-Ib (5–6 years)	Right femur	Seventeenth–nineteenth century CE	In the ground of the church cemetery

Bone samples examined for this study. 1 = negative control*, 2 = positive control*, 3–6 = ancient human bone samples. * = with permission of the pathological-anatomical collection of the Natural Museum Vienna in the Narrenturm (“Fools’ Tower”).

### Western Blots

#### Treponema pallidum

**Fig. 3:**
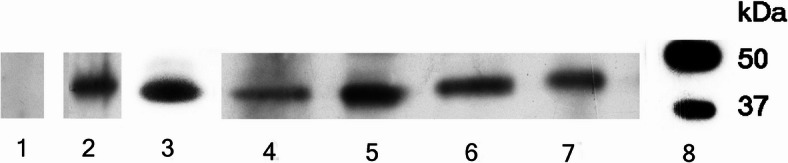
Identification of the 47 kDa protein of *Treponema pallidum*. Western blot with extracellular matrix proteins of ancient human bone. Polyclonal first antibody against *Treponema pallidum* 47 kDa protein, second antibody HRP anti rabbit. Lane: 1: Ind. NM 18,560 negative control of syphilis (Table 1, 1). Lane 2: Ind. NM 1582, positive control of syphilis (Table 1, 2). Lane 3: recombinant 47 kDa protein from *Treponema pallidum*. Lane 4: Ind. E4 from Klosterneuburg, Lower Austria (Table 1, 3). Lane 5: Ind. E5 from Zellerndorf, Lower Austria (Table 1, 4). Lane 6: Ind. E6 from Klosterneuburg, Lower Austria (Table 1, 5). Lane 7: Ind. Tas. 205 from Tasdorf-Rüdersdorf, Brandenburg Germany (Table 1, 6), Lane 8: the molecular weight marker. In all four ancient human bones, the 47 kDa protein from *Treponema pallidum* could be identified. The blots were cropped (see original images in Supplementary Fig. 3).

After infection with *T. pallidum*, the outer membrane of the bacterium has intensive contact with the host. Most of the components responsible for the virulence of the bacterium are located in the outer membrane. Inflammatory effects of *T. pallidum* are primarily mediated by membrane lipoproteins, strong immunogens are the lipoproteins 47 kDa, 17 kDa and 15 kDa^22^. In several tests, the typical *T. pallidum* proteins were identified with comparable results. The 47 kDa protein from *T. pallidum c*ould be reliably detected in all four individuals (Table 1, 3–6; Fig. 3, lane 4–7).

**Fig. 4:**
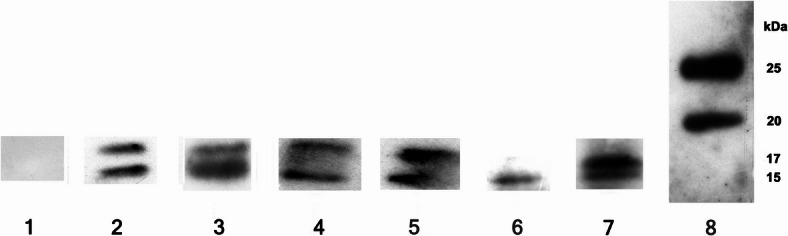
Identification of the 15kda and 17 kDa-proteins of *Treponema pallidum.* Western blot with extracellular matrix proteins of ancient human bone. Monoclonal first antibody against 17 kDa and 15 kDa proteins of *Treponema pallidum*, second antibody HRP anti mouse. Lane 1: Ind. NM 18,560 negative control of syphilis (Table 1, 1). Lane 2: Ind. NM 1582, positive control of syphilis (Table 1, 2). Lane 3: the recombinant proteins 17 kDa and 15 kDa from *Treponema pallidum.* Lane 4: Ind. E4 from Klosterneuburg, Lower Austria (Table 1, 3). Lane 5: Ind. E5 from Zellerndorf, Lower Austria (Table 1, 4). Lane 6: Ind. E6 from Klosterneuburg, Lower Austria (Table 1, 5). Lane 7: Ind. Tas. 205 from Rüdersdorf, Brandenburg Germany (Table 1, 6). Lane 8: the molecular weight marker. In three ancient bones (E4, E5 and Tas. 205) the both typical marker proteins 17 kDa and 15 kDa from *Treponema pallidum* could be identified, in E6 only the 15 kDa protein from *Treponema pallidum* could be detected. The blots were cropped (see original images in Supplementary Fig. 4).

The lipoproteins of *T. pallidum* with a molecular weight of 17 kDa and 15 kDa, could be detected in individuals E4 and E5 (Table 1, 3–4; Fig. 4, lane 4 and 5) and in the child from Tasdorf (Table 1, 6; Fig. 4, lane 7). In the individual E6 (Table 1, 5; Fig. 4, lane 6) the 15 kDa protein was detectable. In the human bone sample from the individual with a known diagnosis of syphilis (Table 1, 2), the marker proteins for treponematoses of 47 kDa (Fig. 3, lane 2), 17 kDa and 15 kDa are detectable (Fig. 4, lane 2). We also tested as a negative control a human individual who did not show any morphological features of syphilitic disease (Table 1, 1). None of these typical marker proteins could be detected (Fig. 3, lane 1; Fig. 4, lane 1). As positive test controls, the recombinant marker proteins TpN47, TpN17 and TpN15 of *T. pallidum* could be identified (Fig. 3, lane 3; Fig. 4, lane 3).

According to the biochemical criteria, all four individuals examined developed treponemal disease. The four individuals presented here were definitely infected with *T. pallidum*. The three E4, E5, and E6 have acquired treponemal disease; the mother probably infected Tasdorf’s child during pregnancy, i.e. congenital treponemal disease.

## Discussion

After the death of a human individual, cell structures such as organelles, membranes and all molecules inside of the cells such as DNA, proteins and lipids are broken down relatively quickly. The degradation products of the cells can randomly accumulate on the bone matrix due to their affinity for hydroxyapatite, in the same way as degraded DNA, which comes from mitochondria and nuclei of cells^23^. For the DNA it does not matter whether it comes from the bone cell or from cells of other tissues, since the DNA sequence is the same as in all cells of the organism. The protein pattern of all living beings differs significantly from tissue to tissue. Furthermore, the protein pattern is also dependent on the current metabolic state. The same principle also applies to impurities, which, via external diagenesis, affect the decomposing organism, and, in particular, the skeletal system. It is therefore important to consider which proteins should be detected before starting an experiment in which archaeological compact bone samples should be processed. The principle of our test is: 1) Proteins, which only weakly adhere to the bone matrix after the death of the individual, accumulate in the first two liquid phases, and must be removed in the course of processing. 2) The experiment continues with the solid substance, which contains ECM proteins that are still tightly bound to hydroxyapatite or collagen^24^. The prerequisite for the successful solubilization of bone NCPs in the solid substance is the loosening of the protein binding to hydroxyapatite or collagen. This can be achieved enzymatically using the enzyme collagenase^13^. The basic conditions must be tested individually for each bone, for example, the amount of bone powder, of collagenase and of the time required. The good coordination of these three factors enables the solubilization of the of ECM proteins of human recent and ancient bone with Laemmlie puffer^25^.

Venereal syphilis is a sexually transmitted infection caused by the bacterium *T. pallidum pallidum*. In modern medicine serology is still the most reliable method for laboratory diagnosis of syphilis regardless of the stage of infection. In the early phase before antibody formation, the pathogen is detected by PCR or dark field^26^. The later stages of syphilis are diagnosed by serological tests, as for example enzyme immunoassay or chemiluminescence assays^27^. The outer membrane of *T. pallidum* contains few proteins minimizing surface-localized antigenic targets recognized by host antibodies or immune cells. The strongest inflammatory effects of *T. pallidum* primarily mediated by the 15 kDa, 17 kDa, and 47 kDa lipoproteins^22,28^. The 47 kDa-protein is highly immunogenic and activates endothelial cells; 17 kDa and 15 kDa proteins induce mainly antibody responses^29^.

The previously described surface changes of the three tibiae from the two charnel houses of Lower Austria are less common in non-specific bone inflammations, in which more irregular surface changes are usually present, since bone remodeling usually occurs faster and more dramatically^30^. In thin-ground sections of the human tibiae (Table 1, 3–5), the typical features of non-gummatous treponematosis, POs and GS (Fig. 2) were microscopically proven^31^. Based on these findings and the absence of cloacae, fistulae, involucrum and sequestrum, the presence of nonspecific osteomyelitis is unlikely and suggests a specific bone inflammation such as treponematosis^32^. GS is not found to this extent in non-specific osteomyelitis and the pronounced formation of polsters clearly indicates a slowly growing inflammatory process as is characteristic of treponematosis in Europe^31,33,34^ (Fig. 2). Polsters consist of so-called agate bone^35^. This explains the slow growth and spreading of this periosteal process^30^. The 5- to 6-years-old child from Tasdorf (Table 1, 6) is a rare case of congenital syphilis in the form of a mineralization disorder of the first molar (mulberry molar)^21^. The diagnosis was confirmed proteomically.

All three typical marker proteins of *T. pallidum* were identified using special antibodies in the four individuals with probable treponemal disease.

If the results of the examined proteins from human ancient bone show the same protein pattern as those of the control proteins in the Western blot, then the specificity of the results is confirmed^36^.

Meanwhile, it has become widely accepted that proteins in ancient human bone are comparatively more stable against diagenetic degradation than aDNA (e.g.,^37,38^). However, what some scholars fail to consider, is that well-preserved proteins, whether recent or ancient, are degraded immediately after removal from their protective tissue [39]. Without minimal protective measures such as cold storage and the addition of protease inhibitors it is not possible to conserve intact proteins^39^.

Shaw and co-worker^40^ processed ancient teeth using the authors’ method of pulverization and extraction of ancient proteins and detected intact antibodies with pathologies related to Paget’s disease and rheumatoid arthritis.

Western blot is regarded as an effective diagnostic tool that is widely used in the clinical settings due to its applicability in direct specific protein identification from a mixture of proteins (e.g.,^41,42^). Of course, the conditions for examining archaeological bone using Western blot analysis are principally the same as for recent samples from hospitals. However, the latter are not expected to show degradation by diagenesis, which makes investigations easier. Since bone collagen is excellently preserved in our samples of the archaeological skeletal remains (Fig. 2), the risk of degradation of the ECM proteins should be comparatively low.

In most publications dealing with the detection of ECM proteins, the investigations were carried out using mass spectrometry (38). To our knowledge, no results have been presented so far using Western blot analysis of archaeological skeletal remains to diagnose treponematoses/syphilis.

As a rule, many typical human bone proteins with the correct molecular weight should be identifiable in a mixture of collagenous and non-collagenous proteins from well-preserved human bones, some of which are thousands of years old. This must be ensured by controls^7^. Prerequisites for good results are processing only the pellet (solid fraction, see methods section) and taking appropriate measures to prevent protein degradation starting during the pulverization phase.

Thus, we can present three cases of acquired syphilis in the individuals from Austria, and one case of congenital syphilis in the child from Tasdorf. In a congenital syphilis case, the infection was transmitted from the mother to the child during pregnancy.

The definitive diagnostic evidence of an infectious disease in compact bone is to identify specific proteins from the bacteria that have infected the organism. Now, the isolated marker proteins 47 kDa, 17 kDa and 15 kDa of *T. pallidum* could be identified by chemiluminescence assays also in archaeologically recovered human bone. This opens the possibility to reliably study the isolated marker proteins of *T. pallidum* in ancient, well-preserved human bones to obtain clues about the history of treponematoses. Of particular interest to the history of treponematoses is the detection of these diseases in the Old World before the discovery of America in 1492 by the Spaniards. The results of a paleoproteomic study could help clarify some facts in the debate about the origin of syphilis. The review by Baker and co-workers provides a good overview on this subject^43^.

In other publications, syphilis was diagnosed in ancient human individuals using immunochemistry or amplified DNA. G. Fornaciari immunohistochemically identified the cytoplasmic filaments of *T. pallidum* in soft tissues of a mummy, which lived in the sixteenth century CE^44^. A purified IgG fraction from a femur of a 200-years-old skeletal specimen from the Easter Island were tested in enzyme-linked-immunosorbent assay (ELISA) against a *T. pallidum* antigen, a significant binding of treponemal activity was measured^45^. C.J. Kolman and co-workers also extracted fragments of the 15 kDa lipoprotein gene from skeletal remains from Easter Island^45^. Fragmented 15 kDa lipoprotein genes were successful identified in European post-Columbian neonates^46^. V. J. Schuenemann and co-workers published the first reconstruction of three *T. pallidum* genomes from the skeletons of one neonates and two infants recovered from the Convent of Santa Isabel in Mexico of the time of seventeenth to the nineteenth century CE^47^. The Schuenemann group presented also exiting results, e.g., treponemal genomes from approximately 2000-years-old human remains from Brazil^48^.

As described in detail by Naba^49^, the ECM of recent tissues is a reservoir for disease-related proteins. We can confirm this statement through our studies with ECM of ancient human bones, even after thousands of years, the protein disease markers can still be detected^12,13,15^.

Depending on the metabolic situation, the ECM protein pattern of the bone is subject to a constant change, which is referred to as bone remodelling^50^. Abnormal ECM remodelling can cause many pathological conditions^51^. During the pathological remodelling of the ECM, excessive amounts of tissue- and pathology-specific turnover products are generated, which are released and transported via partially newly formed transcortical vessels^52^. Often the turnover products contain post-translationally modified (PTM) proteins^53,54^. These modified structures are the main candidates for biochemical marker development, as they may be more related to pathogenesis than unmodified proteins^55^. The PTMs of human bone ECM proteins are not predicted in the sequence of DNA or mRNA^53^. Turnover ECM proteins with PTMs comprise most of the disease biomarkers^54^.

### Critical comments

The causes of diagenesis in archaeological skeletal remains recovered from burials are diverse and can be divided into inanimate and animate agents. The former group includes, for example, mechanical, i.e., physical or chemical forces of the soil and also water. The latter group includes, among others, plant roots, arthropods (e.g., insects, arachnids), worms, small snails, but above all, microorganisms such as bacteria, fungi, and algae. The inorganic components of bone (e.g., bone apatite) can be dissolved by the minerals of the soil and of the exposure to water and are ultimately often completely destroyed. The living agents feed on the organic components of the bone, such as bone collagen (which is also the largest extracellular matrix protein in the bone in terms of volume and quantity). The Vestiges of postmortem damage up to the complete dissolution of the bone tissue in the sense of complete diagenesis can be detected microscopically, classified and attributed to the individual agents^11^.

These postmortem processes can falsify results by affecting the identification of ECM-proteins. The vestiges of these agents of diagenetic destruction are observable in the microscope using plane and polarized light^56^. Several examples of ancient bones which were poorly preserved, and showing various changes due to diagenesis, were also tested from us by our extraction procedure. In these cases, the results showed no protein bands in the silver-stained extraction profiles. The products of these degradation processes (hundreds or even thousands of years old) are very much smaller than 10 kD^57^.

Mass spectrometry is an extremely powerful analytical method that is now widely used in paleoproteomics, particularly for identifying peptides in a protein mixture. The efficiency of ionization determines the sensitivity and accuracy of the measurement. With very small mass differences, it can be difficult to clearly separate individual components. Data evaluation depends on existing databases; chemical modifications of ancient human bone samples have mostly not been documented yet^58^. Unlike Western blot analysis, mass spectrometry cannot specifically detect a single protein in a targeted manner, since usually a large number of proteins or protein fragments are detected simultaneously^38^.

Electrophoretic separation of ECM-proteins from ancient bone followed by Western blot analysis is well suited for identifying individual target proteins using specific antibodies. Several preliminary tests are usually necessary to optimize electrophoresis and blot analysis for the target protein. The solubility of the protein mixture is crucial for the result. Electrophoretic detection of ECMs requires a comparatively large amount of time due to several work steps, all of which must be carried out very carefully and, of course, especially under cold and sterile conditions.

aDNA is highly susceptible to contamination by endogenous molecules, further enhanced by the amplification process. The ECM proteins are protected by the mineralized bone matrix even after the individual’s death; the aDNA is degraded, and the aDNA fragments are loosely attached to the bone, foreign DNA can also attach, which further complicates the methodology. However, aDNA cannot answer all questions; the detection of ECM proteins may be more suitable for clarifying immune processes; aDNA has provided very valuable genetic information about human history^59^.

## Conclusions

Our results demonstrate that NCPs in well-preserved ancient human compact bone are still firmly anchored to hydroxyapatite and collagen even after hundreds of years. Our improved solubilization technique of the NCPs in the ECM of ancient human bone increases the chance of identifying additional protein markers of other infectious diseases or tumours. The problem of establishing a reliable diagnosis using macroscopic, microscopic and biochemical methods is illustrated using ancient skeletal remains of three cases of acquired non-gummatous syphilis and one case of congenital syphilis. The diagnosis of treponematosis was established by the detection of the marker proteins of *Treponema pallidum*, the lipoproteins 47 kDa, 17 kDa and 15 kDa. The results demonstrate that, in the course of a paleopathological study, biochemical examinations in conjunction with microscopic diagnostics establish reliable diagnoses.

## Materials and methods

### Sampling

For the proteomic, macro- and micromorphological analyses, ancient human bone samples were taken from very well preserved long bones of four individuals excavated from two Austrian and one German archaeological sites: Table 1, 3-6. Unfortunately, the bone sample could only be dated archaeologically.

Also processed were a bone sample from a recent human with known diagnose of syphilis (NM 1582) and a bone sample from a recent human without syphilis (NM 18,560). Both bone samples came from a historical skeletal collection housed in the Federal Pathological Museum in Vienna (permission was granted in 2014) dating to the nineteenth and beginning twentieth century (Table 1, 1-2).

### Separation of bone samples for the biochemical investigation

After cleaning, degreasing and powdering of the compact bone samples (Table 1), we get bone powder I, after extraction process with 4M Guanidin-HCl the result is bone powder II^7^. Bone powder II can be stored for years at − 20 °C and is ready for solubilization of the NCPs in the ECM of bone.

### Solubilization of ECM-proteins^13^

Shortly described, based on the principle of an enzyme test, *Clostridium histolyticum* collagenase (IUBMB Enzyme Nomenclature EC3.4.24.3, NB-8, Nordmark, Uetersen, Germany) is a proteolytic enzyme that cleaves the peptide bonds in the triple helix from collagen in situ. This enzyme loosens the matrix and allows NCPs to dissolve, protected by inhibitors against proteases. The ratio of bone powder (10^−3^ g) to collagenase (approx. 10^−3^ U), time (5–30 min, 37 °C) must be tested for each different human bone powder-II.

All procedures were performed with gloves, autoclaved instruments, and autoclaved or sterile filtered solutions.

### Electrophoresis according to Laemmlie 1970^25^

The dissolved ECM proteins of the bone were heated at 95°C for 5 minutes, cooled, centrifuged at 15,000×*g*, the supernatant applied to the SDS-PAGE. The recombinant proteins TpN15, TpN17, and TpN47 *T. pallidum* (Firm MyBiosource San Diego CA, USA) were used as test controls.

The three lipoproteins of *T. pallidum* were detected with two different polyacrylamide-concentrations; 11.5 % T, 2.5% C (for the 47kDa lipoproteins) or 13.5% T, 2.5% C (for lipoproteins 17kDa and 15kDa). The gel runs with cooling in a Hoefer Electrophoresis Unit (SE 260, CA, USA). The gels were further processed by Western blot.

### Western blot analysis

The separated human ECM-proteins in the gel are electro-transferred in a wet blot transfer unit (Hoefer TE22, CA, USA) to PVDF membrane (Millipore, CA, USA) the duration depends on the molecule to be identified. Blocking of non-specific binding sites takes place with starting block-buffer (Firm Perbio Science, Germany). Incubation is performed with one of the following primary antibodies: anti *T. pallidum* polyclonal (OriGene Technologies Rockville, MD, USA) and anti *T. pallidum* monoclonal (firm immunological and biological test system ibt, Binzwangen, Germany).

Horseradish peroxidase-linked anti-rabbit (Dianova, Hamburg, Germany) or horseradish peroxidase linked anti-mouse (Bio Rad, Europe) was used as secondary antibody. Bands were visualized using an enhanced chemiluminescence detection (Millipore, CA, USA) system. Between the different steps, the washing procedure with phosphate-buffered saline/tween 20 (PBS-T) was conducted.

Before the actual series of tests for detecting disease markers, preliminary tests were performed on the samples. After SDS-PAGE, silver staining was used to determine which solubilization conditions for the proteins produce good band patterns. If the normal bone proteins osteonectin and osteopontin (as well as control proteins) can then be identified in the Western blot, the appropriate test conditions for the disease markers are also present.

Minimum rules for correct Western blotting are well compiled in the work of Gilda and co-workers^60^.

### Light microscopic techniques

Small samples were sawn out of the corresponding bones of the tibiae of the three adult individuals from Austria listed in the Table 1. Thin-ground sections examined in plain and polarized transmitted light were prepared for microscopic analysis according to the protocol of Schultz and Brandt^11^. The microscopic diagnosis was established based on the presence of PO and GS^11^.

## Electronic supplementary material

Below is the link to the electronic supplementary material.

Supplementary Material 1

Supplementary Material 2

Supplementary Material 3

Supplementary Material 4

Supplementary Material 5

Supplementary Material 6

Supplementary Material 7

Supplementary Material 8

Supplementary Material 9

Supplementary Material 10

Supplementary Material 11

Supplementary Material 12

Supplementary Material 13

## Data Availability

The data that support the findings of this study have been deposited in the Department of Anatomy and Cell Biology of the University Medical Center Goettingen, Germany, and are available from the corresponding author upon request.
