# Supplementary material for: Identification of typical marker proteins of Treponema pallidum in compact human bone using morphological and biochemical techniques
Source: Sci Rep. 2025 Aug 6;15:28743. doi: 10.1038/s41598-025-12970-z (PMC12328630; doi:10.1038/s41598-025-12970-z)
Supplement: Supplementary file 13 — Supplementary Material 13 [file 41598_2025_12970_MOESM13_ESM.docx]

**Fig. S3a**

Lane 1: neg. control NM 18560 no 47kDa protein

Lane 2: recombinant protein 47kDa *T. pallidum*

Lane 3: molecular weight marker

**Fig. S3b**

Lane 1: positive control NM 1582, 47kDa protein

Lane 2: molecular weight marker

Lane 3: recombinant protein 47kDa *T.pallidum*

**Fig. S3c**

Left lane: molecular weight marker

Lane 1: recombinant 47kda protein *T. pallidum*.

**Fig. S3d**

Lane 1: 47kDa protein, E4, Klosterneuburg, Lower Austria

Lane 2: 47kDa protein, E5, Zellerndorf, Lower Austria

Lane 3: 47kda protein, E6, Klosterneuburg, Lower Austria

Lane 4: 47kDa protein, Tas 205, Tasdorf-Rüdersdorf, Germany

T.P.Ko: recombinant 47kDa protein of *T. pallidum*

**Fig. S4a**

Lane 1: molecular weight marker

Lane 2: negative control NM 18560, no 15kDa protein, no 17kDa protein

Lane 3: recombinant proteins 47 kDa, 17kDa and 15kDa proteins of *T. pallidum*.

**Fig. S4b**

Lane 1: positive control NM 1582, 15kDa protein and 17kDa protein

Lane 2: molecular weight marker

**Fig. S4c**

Left lane: molecular weight marker

Lane 1: recombinant 15kDa, 17kDa proteins of *T. pallidum*

**Fig. S4d**

Left lane: molecular weight marker

Lane 1: E4 Klosterneuburg, Lower Austria, 15kDa protein and 17kDa protein

**Fig. S4e**

Lane 1: E5 Zellerndorf, Lower Austria 17kDa protein and 15kDa protein

Right lane: molecular weight marker

**Fig. S4f**

Left Lane: molecular weight marker

Lane 1: E6 Klosterneuburg, Lower Austria 15 kDa protein

**Fig. S4g**

Left lane: molecular weight marker

Lane1: Tas 205, Tasdorf-Rüdersdorf, Germany, 17kDa protein and 15kDa protein

**Fig. S4h**

molecular weight marker
